# First in situ 3D visualization of the human cardiac conduction system and its transformation associated with heart contour and inclination

**DOI:** 10.1038/s41598-021-88109-7

**Published:** 2021-04-21

**Authors:** Tomokazu Kawashima, Fumi Sato

**Affiliations:** Department of Anatomy, School of Medicine, Toho University, 5-21-16 Omori-Nishi, Ota-ku, Tokyo, 143-8540 Japan

**Keywords:** Anatomy, Cardiology

## Abstract

Current advanced imaging modalities with applied tracing and processing techniques provide excellent visualization of almost all human internal structures in situ; however, the actual 3D internal arrangement of the human cardiac conduction system (CCS) is still unknown. This study is the first to document the successful 3D visualization of the CCS from the sinus node to the bundle branches within the human body, based on our specialized physical micro-dissection and its CT imaging. The 3D CCS transformation by cardiac inclination changes from the standing to the lying position is also provided. Both actual dissection and its CT image-based simulation identified that when the cardiac inclination changed from standing to lying, the sinus node shifted from the dorso-superior to the right outer position and the atrioventricular conduction axis changed from a vertical to a leftward horizontal position. In situ localization of the human CCS provides accurate anatomical localization with morphometric data, and it indicates the useful correlation between heart inclination and CCS rotation axes for predicting the variable and invisible human CCS in the living body. Advances in future imaging modalities and methodology are essential for further accurate in situ 3D CCS visualization.

## Introduction

Correct anatomical mapping is fundamental and crucial for biomedical sciences. Current advanced imaging modalities with applied tracing and processing techniques allow high-quality visualization of almost all internal structures and regions of interest in the human body^1–6^. However, one of the few in situ structures invisible in 3D is the human cardiac conduction system (CCS), due to its minute size and deep localization, and lack of a specific tracer for in situ CCS imaging. The current clinical imaging modalities cannot visualize the CCS morphology in living patients.

The CCS produces and transmits electrophysiological impulses for regulating the coordinated heart contractions, and CCS disorders cause cardiac arrhythmia. Therefore, in situ CCS mapping can provide accurate anatomical information, improve the understanding of the normal and abnormal conduction mechanisms, and help in evolving better strategies for improving pacing and ablation outcomes. This may contribute to the prevention of new-onset postoperative conduction disorders^7–11^.

As histologically identified and analyzed, the CCS is a minute structure hidden in the sub-epicardium, fibrous skeleton, and myocardium. The macroscopic structure is difficult to visualize in authentic in-body images. Instead, in clinical anatomical papers and atlases of the heart it is commonly presented by superimposing a CCS illustration on an actual cardiac photograph^12–14^. Some recent detailed anatomic studies have overcome this difficulty based on the histological criteria for macroscopic identification^15–21^. However, while presenting a minute CCS dissection and a real image, they also highlighted the technical limitations of the dissection approach of the atrioventricular (AV) conduction axis. For example, the left bundle branch was shown as the left chamber view, and the other AV conduction axis as the right chamber view. In this context, imaging the CCS from any direction is difficult because of a limited 2D photographic presentation depending on the dissection.

For a 3D histological reconstruction of the human CCS, it is technically difficult to take serial sections of the entire heart due to its large size. A few small studies attempted reconstruction by targeting a limited area of the adult CCS or a more extensive area of the fetal or infant CCS^7,19,22–24^. In some studies, morphometric human CCS data are also very valuable for size estimations. Since all histologic examinations involve deformation and shrinkage of tissue samples based on the tissue types and sample preparation processes^25–27^, it is also necessary to verify the size differences between 3D macroscopic and histological measurements of CCS components.

Recent 3D technological advances have enabled visualization of the human CCS by contrast-enhanced computed tomography (CT) imaging techniques^28–32^, in which the specimen is immersed in a stain (mostly iodine) to detect a few differences from the surrounding working myocardium. With a small area of focus required for micro CT generally used in this method, scanning a large sample such as the entire adult human heart would be difficult; thus, a smaller sample is needed to obtain a better staining contrast.

Despite these technical difficulties, there have been some successes. Atkinson et al. (2016) created a 3D histological reconstruction of the entire CCS using an intact fresh human heart^7^, and Stephenson et al. (2017) also visualized the 3D architecture of the entire CCS using contrast-enhanced micro CT on four fresh human hearts^30^. These articles elucidated the analytic approach to the 3D architecture of the adult human CCS ex vivo; however, the actual arrangement within the body remains unclear.

In contrast to the limitations in 2D photographic presentation of physical dissection and the significant shrinkage and deformation in 2–3D histology described above, advances in imaging tools allow us to change the transparent of and/or cut structures other than the region of interest, to visualize segmentation from any direction without morphometric issues in processing, and to transfer data for computer-based analysis. This is currently one of the leading morphological tools and is also known as “virtual dissection”^1,2,5,6,20,23^. With some challenges imposed by our formalin-fixed samples from the elderly, we conducted a visualization of the 3D CCS morphology by combining detailed micro-dissection with the advantages of virtual imaging dissection. The next step is to understand the 3D arrangement within the adult body and also the four-dimensional (4D) morphology, in which another dimension such as a time, a specific function, or a pathology dimension is added. Revealing the in situ arrangement of the human CCS associated with the individual positional changes in the normal heart is the first task before analyzing its functional or pathological changes.

The main goals for this study of the adult human CCS were: (1) to visualize the actual 3D internal CCS arrangement from the sinus node (SN) to the bundle branches within the body and to provide morphometric data about its individual variation; and (2) to elucidate the 3D CCS transformation from the standing to the lying down position of the heart as an additional dimension.

## Results

### In situ 3D visualization of the human CCS

**Figure 1:**
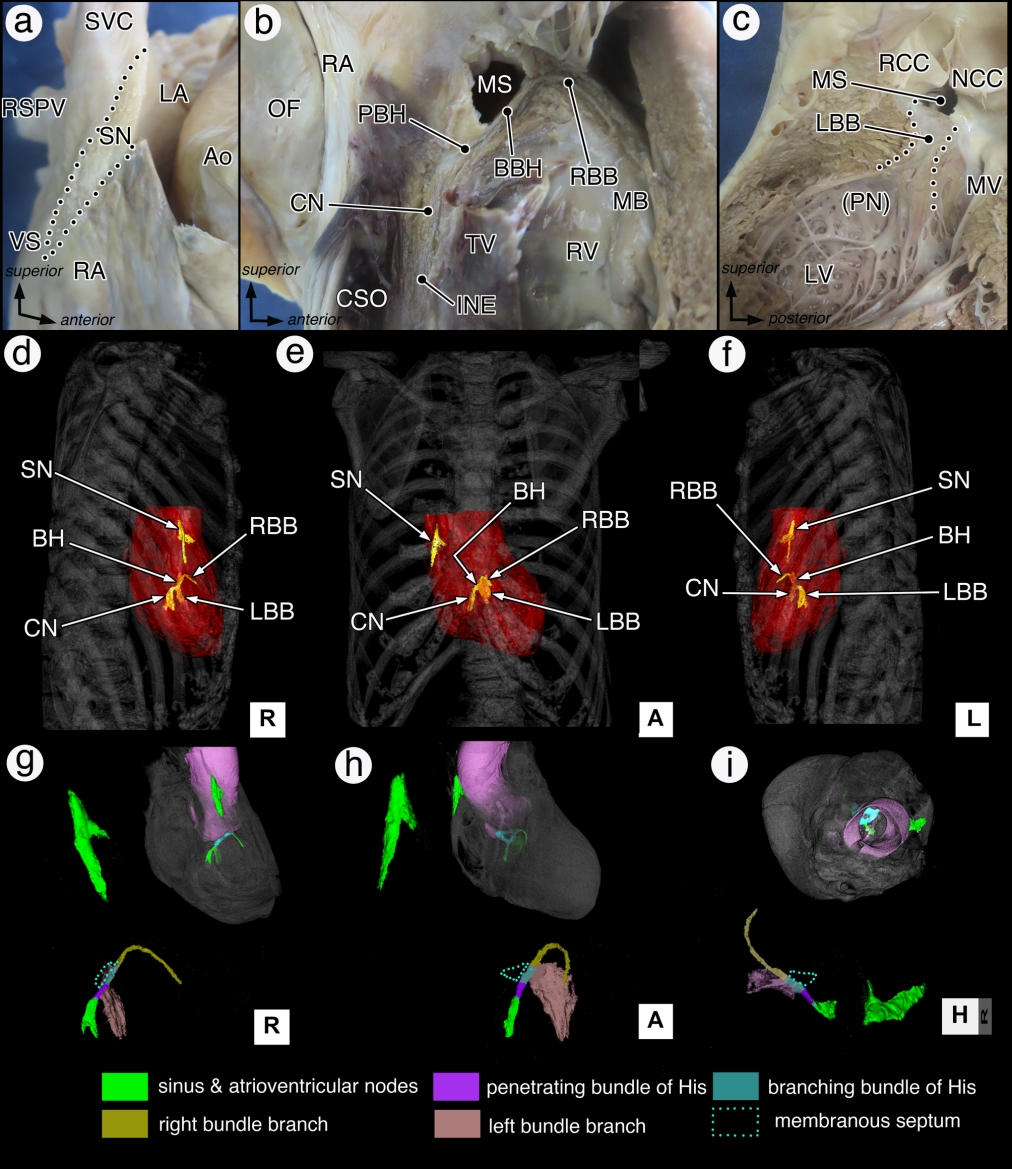
Physical and virtual dissection of the human CCS from the sinus node to the bundle branches in the standard oblique heart. The physical dissection of the sinus node situated at the terminal groove in the right anterior oblique view (**a**), the atrioventricular conduction axis from the compact node to the right bundle branch in the right chamber view (**b**), and the left bundle branch in the left chamber view (**c**). The virtual dissection of the three-dimensional (3D) architecture of the CCS and its segmented components within the body in the right lateral (**d,g**), frontal (**e,h**), left lateral (**f**), and superior (**i**) views, respectively. *Ao* aorta, *BBH* branching bundle of His, *CN* compact node, *CSO* coronary sinus orifice, *INE* inferior nodal extension, *LA* left atrium, *LBB* left bundle branch, *LV* left ventricular, *MB* moderator band, *MS* membranous septum, *MV* mitral valve, *NCC* non-coronary cusp, *OF* oval fossa, *PBH* penetrating bundle of His, *PN* Purkinje network, *RA* right atrium, *RBB* right bundle branch, *RCC* right coronary cusp, *RSPV* right superior pulmonary vein, *RV* right ventricle, *SN* sinus node, *SVC* superior vena cava, *TV* tricuspid valve, *VS* venous sinus.

To visualize the CCS at a macroscopic level, we developed a minute physical dissection analysis using formalin-fixed elderly human cadavers based on the histological criteria for CCS identification (see details in methods) and combined it with virtual CT dissection as shown in Fig. 1. This technique allowed the first in situ 3D visualization of the human CCS from the SN to the bundle branches, from any direction independent of the dissection plane, and provided the 3D morphometric data (Fig. 1 and Table 1).

**Table 1:** Dimensions of the CCS components.

	Length	Width	Thickness
Sinus node	33.9 ± 7.8 (20.5–53.5)	7.4 ± 1.9 (4.0–12.3)	1.6 ± 0.4 (1.0–2.4)
Compact AV node	8.7 ± 1.7 (5.0–11.7)	4.4 ± 1.2 (2.4–6.9)	2.2 ± 0.6 (1.4–4.0)
**Bundle of His**	14.3 ± 2.9 (7.0–20.6)	NA	NA
PBH	4.7 ± 1.6 (1.7–7.7)	1.4 ± 0.4 (0.7–2.2)	1.2 ± 0.3 (0.7–1.9)
BBH	9.6 ± 2.7 (5.3–14.9)	1.2 ± 0.3 (0.7–1.7)	1.3 ± 0.3 (0.9–2.2)
**LBB**			
Origin	NA	9.3 ± 2.6 (5.1–14.5)	0.4 ± 0.1 (0.3–0.7)
Bending part*1	NA	11.4 ± 2.7(5.3–15.5)	0.4 ± 0.1 (0.3–0.6)
**RBB**			
Proximal *2	NA	1.1 ± 0.2 (0.8–1.7)	1.1 ± 0.3 (0.3–1.6)
Distal *3	NA	1.0 ± 0.2 (0.6–1.5)	0.9 ± 0.2 (0.6–1.2)

Values are mean ± SD (range) in mm.

*^1^Distal of common faacicle of the LBB coursing bendingly at the left interventricular crest.

*^2^Origin part of the RBB from the end of the BBH.

*^3^Just caudal point of bending and transitional part of the RBB which the ascending RBB bends and then descends.

As in previous reports, internodal pathways could not be recognized morphologically using any histological methods; hence, the CCS was divided and analyzed in two parts: SN and atrioventricular conduction axis (AVCA).

Importantly, these visualization findings clearly emphasized and updated two unresolved issues about the CCS topography. First is the internal placement of the branching bundle of His (BBH). Our visualizations clearly showed the bundle of His ascending obliquely along the anteroinferior margin of the membranous septum (Fig. 1b,g). The second is the bifurcation of the bundle branches from the BBH. Our 3D images clearly showed that the bundle branches do not bifurcate simultaneously from the BBH; instead, the BBH completely branched off the left bundle branch (LBB) like a thin drape and then formed the right bundle branch (RBB) (Fig. 1g–i).

### Correlation between cardiac and CCS inclinations in 2- to 3-dimensions

**Figure 2:**
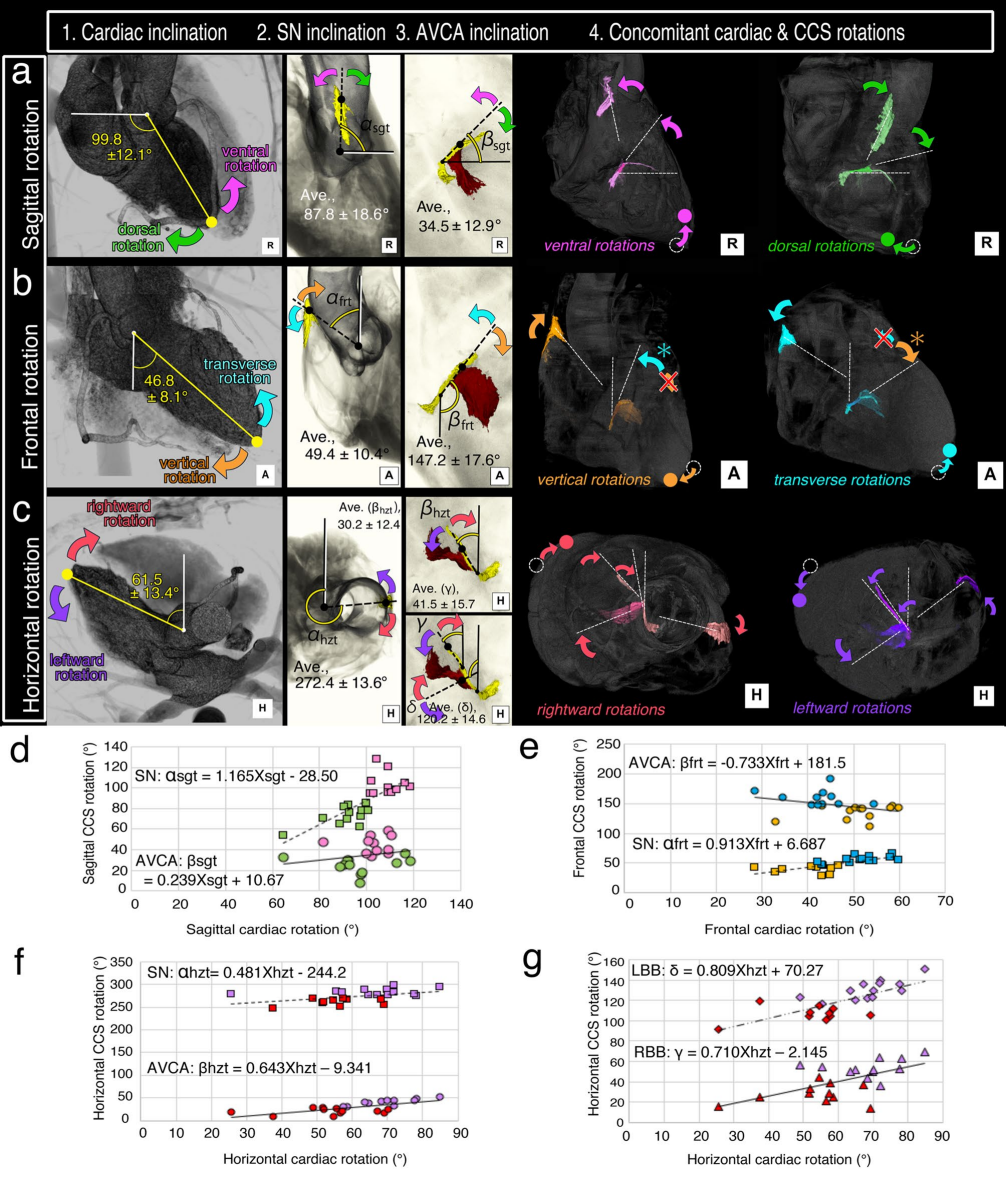
The 2D projection images based on the 3D CCS transformation by cardiac rotation along the 3 axes in the sagittal (**a**), frontal (**b**), and horizontal (**c**) planes of the thorax (**a**–**c**). Each column shows the cardiac inclination/rotation (column 1), the sinus node (SN) inclination (column 2), the atrioventricular conduction axis (AVCA) inclination (column 3), and the concomitant cardiac and CCS rotations (column 4). The colors of the rotating arrows in rows (**a**–**c**) indicate the direction of the cardiac and CCS rotations: pink, ventral rotation; green, dorsal rotation; orange, vertical rotation; blue, transverse rotation; red, rightward rotation; purple, leftward rotation. Scatterplot graphs showing the relationship between angles of cardiac and CCS component rotations in the sagittal (**d**), frontal (**e**)**,** and horizontal rotations (**f,g**) of all examined hearts. The dashed (SN) and solid (AVCA) lines are the lines of best-fit from the linear regression analysis. The scatterplot colors represent the same colors used for cardiac rotations. *AVCA* atrioventricular conduction axis, *LBB* left bundle branch, *RBB* right bundle branch, *SN* sinus node.

To assess the relationship between the CCS and heart inclinations, we first examined the CCS projections in three rotational axes: sagittal, frontal, and horizontal, and their 3D CCS transformations associated with the cardiac inclination changes (Fig. 2a–c). The results of bivariate regression analysis of the relationship between angles of the cardiac and CCS components are shown in Fig. 2d–g.

For evaluation of heart rotation, the reference line connecting the center of the aortic valvar orifice and the left ventricular apex served as the basic cardiac axis (column 1 in Fig. 2 and Table 2). The average reference axis of the 23 study hearts as assessed relative to the sagittal (Fig. 2a1), frontal (Fig. 2b1), and horizontal (Fig. 2c1) thoracic planes were 99.8 ± 12.1°, 46.8 ± 8.1°, and 61.5 ± 13.4° in the right lateral, frontal, and superior (head) views, respectively (column 1 in Fig. 2 and Table 2). These findings indicated that horizontal and sagittal rotations of the heart produce greater individual and positional changes in the heart than do frontal rotations.

**Table 2:** Average inclinations of the heart and CCS components.

	Abbreviation	Angle (mean ± SD)	Presentation
**1. The inclination of the heart**			
Sagittal rotation of the caridac inclination	NA	99.8 ± 12.1°	Figure 2a1
Frontal rotation of the cardiac inclination	NA	46.8 ± 8.1°	Figure 2b1
Horizontal rotation of the caridac inclination	NA	61.5 ± 13.4°	Figure 2c1
The inclination of the conduction components			
**2. Sinus node **(**SN**)			
Sagittal rotation of the sinus node	α_sgt_	87.8 ± 18.6°	Figure 2a2
Frontal rotation of the sinus node	α_frt_	49.4 ± 10.4°	Figure 2b2
Horizontal rotation of the sinus node	α_hzt_	272.4 ± 13.6°	Figure 2c2
**3. Proximal atrioventricular conduction axis **(**AVCA**)			
Sagittal rotation of the proximal AVCA	β_sgt_	31.9 ± 13.2°	Figure 2a3
Frontal rotation of the proximal AVCA	β_frt_	32.9 ± 17.6°	Figure 2b3
Horizontal rotation of the proximal AVCA	β_hzt_	30.2 ± 12.4°	Figure 2c3
**4. Right bundle branch **(**RBB**)			
Horizontal rotation of the right bundle branch	γ	41.5 ± 15.7°	Figure 2c3
**5. Left bundle branch **(**LBB**)			
Horizontal rotation of the left bundle branch	δ	117.1 ± 15.1°	Figure 2c3

The reference line connecting the center of the SN head to the top of the terminal crest and center of the aortic valvar orifice served as the reference axis of the SN (column 2 in Fig. 2). The average SN inclination angles were 87.8 ± 18.6°, 49.4 ± 10.4°, and 272.4 ± 13.6° in the sagittal (Fig. 2a2), frontal (Fig. 2b2), and horizontal (Fig. 2c2) planes, respectively (column 2 in Fig. 2). The relationships between sagittal, frontal, and horizontal cardiac rotations (X_sgt_, X_frt_, and X_hzt_) and those of the corresponding SN rotations (α_sgt_, α_frt_, and α_hzt_) were as follows:

$${\alpha_{sgt}} = \, 1.165{X_{sgt}} - \, 28.50$$

$${\alpha_{frt}} = \, 0.913{X_{frt}} + \, 6.687$$

$${\alpha_{hzt}} = \, 0.481{X_{hzt}} - \, 244.2$$

These results showed that each SN inclination significantly correlated with each cardiac inclination in the sagittal (dorso-ventral), frontal (vertico-transverse), and horizontal (right- and leftward) rotations (Fig. 2d–f).

The reference line connecting the end of the compact AV node and the branching point of the RBB, which is a relatively linear part of the AVCA, served as the reference axis for the AVCA (column 3 in Fig. 2 and Table 2). Since it was difficult to define the measurement point for RBB and LBB, which makes a large curve along the way, only the proximal part—which can be measured as a relatively direct part—was measured in the horizontal plane. The average inclinations of the AV conduction components were: angle (βsgt) of the bundle of His to the horizontal line was 34.5 ± 12.9° cranially in the right lateral view (Fig. 2a3); angle (βfrt) of the bundle of His to the sagittal line was 147.2 ± 17.6° to the left in the frontal view (Fig. 2b3), and angle (βhzt) of the bundle of His to the horizontal line was 30.2 ± 12.4° in the horizontal view (Fig. 2c3). The RBB angle (γ) to the sagittal line was 41.5 ± 15.7° to the left in the superior (head) view (Fig. 2c3), and the LBB branching angle (δ) of the center LBB from the bundle of His to the sagittal line was 120.2 ± 14.6° to the left in the superior (head) view (C). The relationships between the sagittal, frontal, and horizontal cardiac rotations (X_sgt_, X_frt_, and X_hzt_) and those of the corresponding proximal AVCA rotations (β_sgt_, β_frt_, and β_hzt_) were as follows:

$${\beta_{sgt}} = \, 0.239{X_{sgt}} + \, 10.67$$

$${\beta_{frt}} = \, - 0.733{X_{frt}} + \, 181.5$$

$$ {\beta_{hzt}} = \, 0.643{X_{hzt}} - \, 9.341$$

In evaluating distal AVCA inclination, the relationship between cardiac inclination and the bundle branches was determined using only data in the horizontal planes, as setting certain metrics was difficult due to their complex morphologies. The horizontal cardiac rotation (X_hzt_) was also positively correlated with each of the RBB (γ) and LBB (δ) angle rotations as follows:

$$\gamma = \, 0.710{X_{hzt}} - \, 2.145$$

$$\delta = \, 0.809{X_{hzt}} + \, 70.27$$

The measurement angles/inclinations were defined such that the rotational directions of the cardiac and each CCS component axes were concomitant with each other in all rotational planes, but the directions of proximal AVCA and cardiac rotation did not correlate in only the frontal rotation (Fig. 2b). In other words, the proximal AVCA rotated in the opposite transverse direction (counterclockwise, colored in blue) when the heart underwent vertical rotation (clockwise, colored in orange), whereas the proximal AVCA rotated in the opposite vertical direction (clockwise, colored in orange) when the heart underwent transverse rotation (counterclockwise, colored in blue) (Fig. 2b4). In the formula relating the inclination of the heart (X_frt_) and AVCA (β_frt_), inverse proportionality could be clearly reconfirmed: β_frt_ = − 0.7329X_frt_ + 181.52 (Fig. 4e).

**Figure 3:**
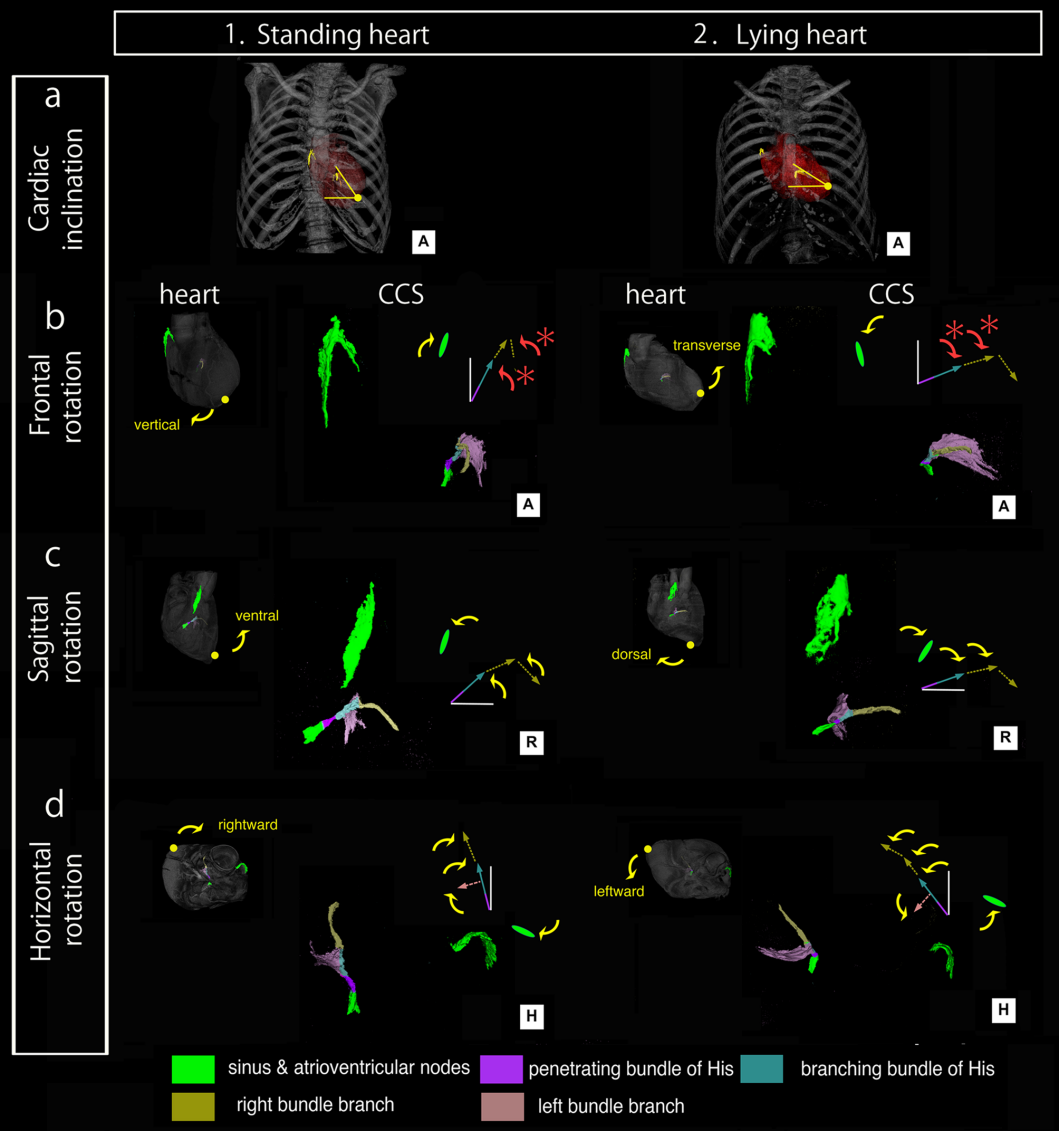
Comparison of 2-3D transformation of the CCS arrangement in the standing (**column 1**) and lying (**column 2**) positions of the heart. The standing position typically exhibits the ventral, vertical, and rightward rotation of the heart, whereas the lying position exhibits the dorsal, transverse, and leftward rotations. Yellow arrows for the heart and CCS are concomitant clockwise or counterclockwise rotations, whereas red arrows of the CCS components marked by asterisks indicate reverse rotation and do not correlate with cardiac rotation in the frontal plane/rotation.

To evaluate the inverse frontal CCS rotation, we next tested whether the 3D CCS changes were associated with the three cardiac rotation axes by comparing 2–3D CCS changes in the standing and lying positions of the heart (Fig. 3). Although anatomic findings for the three cardiac rotation axes—dorso-ventral, vertico-transverse, and left- and rightward—varied individually, our findings for the concomitant cardiac and CCS rotations showed that the standing heart exhibited the ventral, vertical, and rightward rotations (Fig. 3a). In contrast, the lying heart exhibited the dorsal, transverse, and leftward rotations (Fig. 3b). Hence, when the inclination of the heart changed from standing to lying, the SN changed from the dorso-superior to the right lateral position, and the AVCA changed from the vertical to a leftward horizontal position. Furthermore, this comparison of the standing and lying hearts also showed the difference in the range of motion between the two CCS components depending on their surrounding connections: a slight change in the position of the SN, which is located on the terminal groove between the right atrium and superior vena cava, fixed relatively tightly by the pulmonary veins and the superior and inferior vena cava, whereas there was a dynamic change of the AVCA position, which is mainly contained within the flexible ventricles (Fig. 3). We also confirmed that the 2D projection finding of the AVCA rotation direction opposite to that of the frontal cardiac rotation was shared in this comparison analysis (red rotation arrows in Fig. 3).

### 3D CCS transformation from standing to lying hearts: into 4D

**Figure 4:**
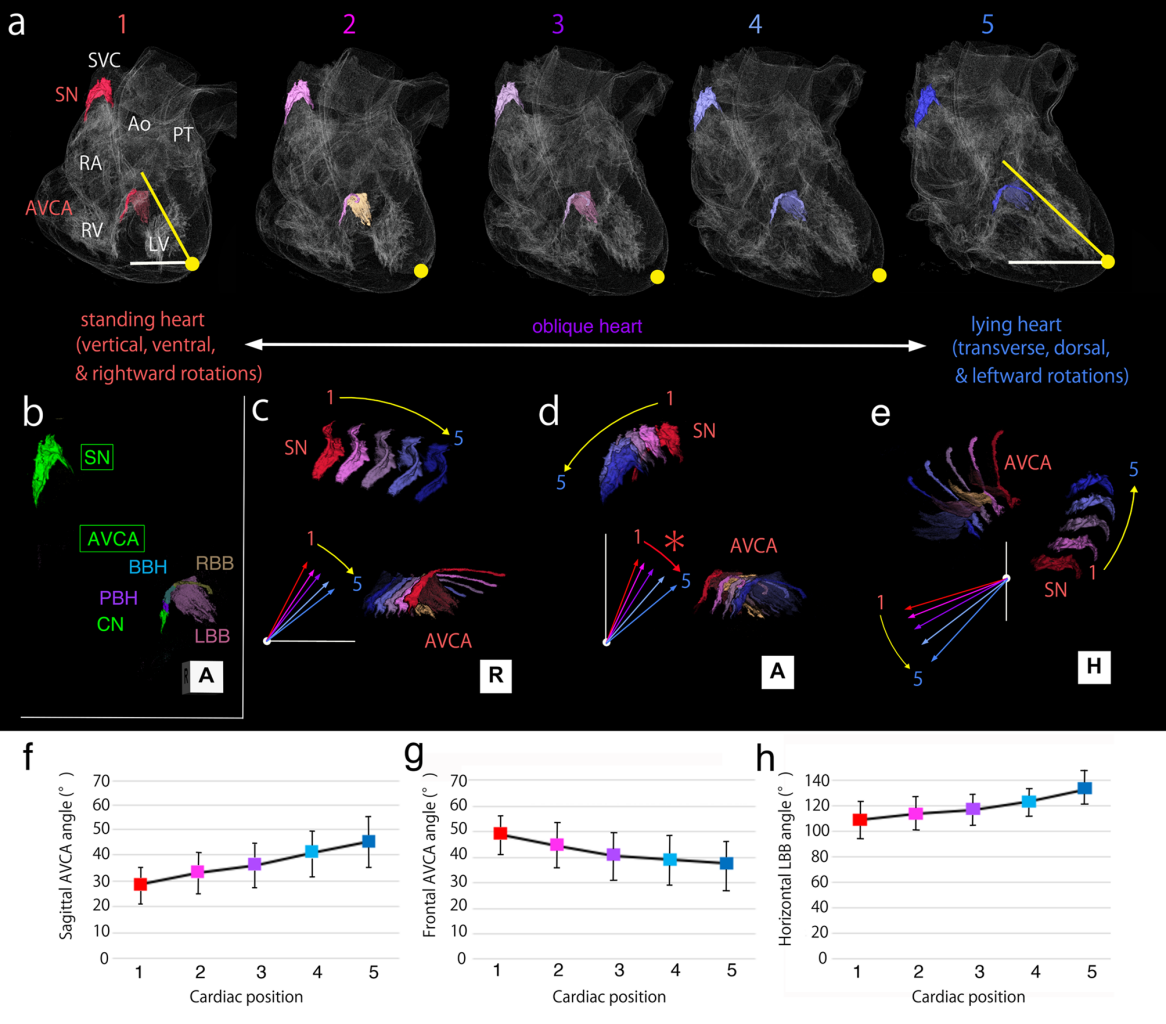
CT imaging-based simulation of the CCS arrangement by gradual stepwise rotations of the heart with standard CCS arrangement from the standing to lying position. The warm to cold colored CCS commonly represent the typical CCSs in the standing (1) to lying (5) hearts, respectively. The upper (**a**) and middle (**b**) rows show the gradual 3D transformation of the CCS within the heart and the CCS itself in the frontal view, respectively. The gradual changes of the superimposed CCS view are shown from the right lateral (**c**), frontal (**d**), and superior (**e**) aspects in the lower row. The graphs also show the changes in the simulation of the 2D projection angles of the CCS in the sagittal (**f**), frontal (**g**), and horizontal (**h**) rotations. As indicated in Fig. 3, the CCS and heart rotations are indicated by yellow rotating arrows when they are in the same rotating direction and by red rotating arrows when rotating in opposite directions. These findings show that the actual and theoretical simulation findings are consistent. Only the frontal cardiac and CCS rotations did not correlate, as shown in the 2D projection findings; however, the stepwise 3D CCS transformation was recognized with cardiac rotation from the standing to lying position. *Ao* aorta, *AVCA* atrioventricular conduction axis, *PT* pulmonary trunk, *RA* right atrium, *RV* right ventricle, *SN* sinus node, *SVC* superior vena cava.

Although the examined samples were strictly selected from normal elders, comparison between individuals cannot rule out the effects of other factors such as potential pathological changes. Hence, to establish stepwise changes in cardiac rotation as the criteria for CCS localization, a simulation of individual CCS transformation was performed using positional changes in rotation from the theoretical standing (ventral, vertical, and rightward rotated) heart to the lying (dorsal, transverse, and leftward rotated) heart in six standard oblique hearts selected from 23 examined hearts in this study (Fig. 4). In this theoretical CT image-based simulation, the three cardiac axes were rotated in a stepwise manner based on the range of changes from the standard oblique (average inclination) to standing (reduced by one standard deviation from the mean in three rotational axes; mean - SD) and lying (one standard deviation added to the mean in three rotation axes; mean + SD) positions. Specifically, each of six standard oblique hearts was rotated along sagittal, frontal, and horizontal axes into five positions: Cardiac position #1, the mean value-2 standard deviations (SD) (Fig. 4a1); Cardiac position #2, the mean-SD (Fig. 4a2); Cardiac position #3, the mean ± 0 (Fig. 4a3); Cardiac position #4, the mean + SD (Fig. 4a4); and Cardiac position #5, the mean + 2SD (Fig. 4a5). When the heart moved from the standing to lying position, the apex showed dorsal rotation, but the transverse and leftward rotations eventually resulted in reverse ventral rotation of the apex. However, the CCS itself showed a series of gradual changes: the SN shifted from the dorso-superior to the right outer side and the AVCA ran transversely to the left. Thus, each heart rotational axis was visualized as a distinct change from the standing to lying position, whereas the CCS clearly showed gradual stepwise changes. Since the SN is fixed by the pulmonary veins, it does not change significantly, as in this simulation. However, the AVCA transformation reflects similar changes; hence, we analyzed the changes in the AVCA components (Fig. 4f–h). These simulation results with gradual stepwise rotations were also consistent with those in 2D projection and 2-3D comparison of inverse frontal CCS rotation. Specifically, if the heart rotates transversely, the AVCA should also rotate counterclockwise to become vertical; however, the AVCA rotated in the opposite direction due to the significant effect of the other two rotation axes.

Based on all our findings, it is possible to estimate the CCS localization and inclination based on the inclination and contour of the heart, such as visualization during standing, oblique, or lying positions.

## Discussion

The current study using our developed methods based on physical and virtual CT image-based dissection combined with histological criteria and reconfirmation provided the first successful 3D visualization of the CCS within the human body and also revealed the 3D CCS transformation by cardiac rotations. This anatomical mapping provides precise information on the invisible CCS localization within the human body. It allows observation and image reconstruction from any direction, a benefit unavailable with the typical 2D image display, which relies on conventional anatomical approaches. Our analysis also identified two misunderstandings about the anatomical CCS localization: (1) The BBH runs along the inferoanterior border of the membranous septum, unlike the previous concept that it runs along the lower edge. Mori et al. (2018) also expected this possibility based on virtual 3D cardiac anatomy^33^; (2) The bundle branches do not simultaneously bifurcate from the bundle of His; the BBH branches widely off the LBB, and finally forms the RBB. This finding is very different from the conventional wisdom that both bundle branches simultaneously bifurcate from the BBH, as seen in the four-chamber view schema and 3D illustration in a systematic review^34^. Thus, our accurate localization and characterization of the human CCS improve understanding of the basis of cardiovascular science. On the other hand, our findings also indicated that the 2D and 3D correspondence between the frontal cardiac and CCS rotations was not intuitive. Combined with our results indicating that horizontal and sagittal rotations of the heart produce greater individual and positional changes than does frontal rotation, it seems that the AVCA rotated in the opposite direction in the frontal 2D projection due to the significant effect of the other two rotation axes, but both the SN and AVCA rotated in the same direction, and gradual stepwise rotations correlated with the cardiac rotation in 3D architecture.

Detailed data for CCS components and their extensive and/or similar specialized myocytes have been described immunochemically in mammals, including humans. These include the paranodal area^22,35^, transitional cells between the AV node and working atrial fibers^36^, consistent connection between the AV node and atrium in the normal human heart^9^, the AV ring with a retro-aortic node in some mammals including humans^23,37,38^, the inferior nodal extensions of the AV node^39^, defined CCS components^23,40^, and detailed extension of Purkinje fibers^41,42^. Macroscopic views of the complex Purkinje network have been shown in fresh specimens using various solutions, inks, and stains, with significant individual variation^41,42^; however, some studies showed only the 3D histological reconstruction of these extensive and/or similar specialized structures in an ex vivo heart, and therefore, in situ arrangements remain unclear. An immunohistological approach was difficult in this study because of the need for formalin fixation to maintain placement in the body. This issue must be addressed using recently described extensive and/or similar specialized myocytes to update our mapping model in future studies.

The current sub-macroscopic anatomical study also provided more suitable morphometric data of the CCS than conventional histology. For CCS components that are invisible from the outer or inner surface of the heart, the morphometric CCS data are valuable for size estimation^21,43–45^. The histological dimensions of the adult human SN^8,43–46^ and AVCA^14,21^ have been defined to accurately identify the boundaries and extensions of the CCS components. However, all histologic measurements were much smaller than those of our macroscopic 3D imaging data with histologic reconfirmation. Previous reliable anatomical and histological findings for SN extension describe that the SN body is located cranially near the superior vena caval orifice, and the tail extends inferiorly below the body toward the inferior terminal crest and Eustachian ridge^8,46^. Based on these descriptions, the previous histological measurements indicate that the size is 10-20 mm in length, considering the length between the bases of the superior and inferior vena cava in adult human hearts. The histological examinations involve tissue sample deformation and shrinkage caused by fixation, dehydration, embedding, and sectioning. Depending on the fixatives, fixation method, tissue samples, sectioning pressure, and speed, even in cryosectioning, the rate of tissue shrinkage differs from minimal to 68.1%^25–27^. However, the previous gross CCS measurements support our data^47^. On the other hand, we must mention that even formalin-fixed cadaver tissues have some shrinkage, though not as much as in histological preparations. In addition, the CCS undergoes significant degenerative changes with aging^44,45^; therefore, this study using formalin-embalmed elder cadavers might show degenerative changes in size. Here, we compared the characteristics of the research methods that revealed the 3D visualization of the human CCS and summarized these in Table 3. In remarkable successes of 3D visualization of the human CCS using fresh human hearts deemed non-viable for transplantation, Atkinson et al (2016) applied 3D histological reconstruction methods^7^, whereas Stephenson et al (2017) applied a contrast-enhanced micro-CT method^30^. They visualized and provided data on the more distal Purkinje fiber network, which was difficult with our methods. In addition, the fresh donor hearts they used had less age-related change and less postmortem tissue degradation than did our elder samples; therefore, the results from their model can be considered excellent. Due to the exceptional materials, they examined only 1 and 4 cases, respectively; hence, individual variations are unknown. In addition, the presented orientation of the ex vivo heart was also the expected approximate position of the heart. In contrast, our study material from formalin-fixed elderly cadavers had limitations in size, such as age-related changes, slight contraction by formalin fixation, and the possibility of postmortem changes, although to less a degree than in the general histological methods. However, our study used the whole human cadavers; thus, it was possible to obtain real images on the in situ 3D CCS morphology, unlike the conventional artificial CCS computer graphics within the body. The physical CCS dissection is easily expected to cause significant transposition of the heart, including the CCS. However, the stiffness of formalin-fixed whole cadavers contributes to the preservation of the initial localization and the relationships of the heart and the surrounding organs. With these characteristics of the formalin-fixed material, we minimized the technical bias in our dissection and retained partial muscular bundle and fibrous tissue attached to the CCS, which was not isolated completely from the working myocardium. Our dissection technique also helped to maintain the CCS localization within the stiffened heart (Fig. 6a–f). Based on these materials and techniques, providing morphometric data on the inclinations of the CCS components and obtaining individual variation data from a large number of specimens rather than non-viable organ donor hearts are the strengths of our present study.

**Table 3:** General characteristics in human CCS structural analysis.

	3D histological reconstruction (Refs.^7,19,22–24^)	contrast-enhanced micro-CT analysis (Ref.^30^)	micro-dissection combined with micro-CT (Present study)
Sample	Fresh/formalin-fixed	Fresh	Formalin-fixed
Postmortem tissue preservation	✓✓/ ✓	✓✓	
Structural preservation in form		✓✓	✓
Segmentation accuracy	✓✓	✓	
In situ arrangement/inclination			✓✓
Sample size collectability	✓		✓✓
Remarks	Tough process	Rare mateiral	Skillful dissection
	Limited area		Aging changes

Consequently, each 3D visualization technique has different characteristics; therefore, we must choose a suitable method for our own purposes and anticipate further advances in methodology, techniques, and devices.

We revealed the actual in situ 3D CCS arrangement in normal elderly human hearts for the first time, which was previously only presented in the 3D architecture of the ex vivo adult human heart. As a result, we anticipate a future study aimed at further understanding the mechanisms of normal and abnormal conduction and the electrophysiology of the heart. This precision ultimately improves patient-specific anatomic navigation during catheter manipulation using the heart region, direction, and the angle relevant to pacing and ablation.

After clarifying the 3D CCS arrangement in humans, research of 4D CCS changes is expected to accelerate with advanced technological progress^48^. 4D CCS changes related to the time axis, such as developmental^23,49–51^, growth and aging^44,45^, and normal versus pathologic^52,53^, can enhance our understanding. However, the topographic CCS changes with various heart inclinations remain unclear. Evaluation of human CCS transformation is difficult because the precise in-body arrangement is unknown, with visualization limited by technical complexity. However, if the CCS transformation can be predicted to some extent based on the heart contour and inclination, this basic information might be very helpful for treating cardiac arrhythmias and preventing conduction complications in the future.

Among several indices for cardiac inclination^54–56^, the center of the aortic valve and left ventricular apex were suitable in this analysis for evaluating the correlation with AVCA changes because the proximal AVCA seemed to be situated in the aortic root complex. Heart rotation based on the current study criteria correlated well with the CCS transformation and is considered a suitable indicator for predicting invisible CCS transformation in the living body. Compared with past cardiac inclination indices, our samples from older adults were more transverse compared to those of young, healthy adults. In this study population, AVCA findings showed more degenerative features, especially in the relative thickness of the bundle branches, compared to those of younger and middle-aged adults. Despite the cardiac axis and aging changes, these findings represent the typical physiologic condition of the CCS and the heart itself, based on the strict selection criteria for this study.

In summary, our first 3D model showing the in vivo arrangement of the human CCS from the SN to the bundle branches clarified the correct orientation of each CCS component. These findings might help improve anatomic navigation for cardiac catheterization in pacing and ablation and may prevent new postoperative conduction disorders. Roughly speaking, our results showed that the CCS transformation could be predicted depending on the heart contour and inclination; thus, this anatomic knowledge is also useful for locating the variable and invisible CCS in the living human body. To predict the localization of an individual CCS in more detail, additional information is required. Due to the remarkable individual variation of the compact node (CN) and PBH localization within the triangle of Koch (Cabrera et al, 2020)^21^, and of PBH and BBH localization to the anteroinferior border of MS (Kawashima and Sasaki, 2005)^16^, the setting of the starting point of the AVCA must be defined. If this information is obtained, it may assist in estimating individual CCS localization based on our current relationship formulae for CCS and cardiac inclinations with morphometric data. In the future, advanced electrophysiological mapping systems will assist cardiologists in predicting CCS localization in living patients based on cardiac contour and rotation data obtained through deep learning using big data analysis of individual CCSs.

## Limitations

Due to various methodological issues, in situ 3D CCS images still remained unclear. Our applied methods and material series in formalin-fixed elderly cadavers may also include several limitations such as age-related degenerative changes, poor traceability of the peripheral CCS, and CCS transformation by dissection. On the other hand, our embalmed human cadavers allowed for time-consuming destructive analysis and preservation of in situ orientation. Considering these technical limitations, and the strengths of our study materials and alternative methods, our modified CCS dissection technique, with its minimal CCS displacement and final histological reconfirmation, is the first to visualize the in situ human CCS localization with individual variations and to provide morphometric data. Advances in future modalities and methods are essential for further accurate in situ 3D CCS visualization.

## Methods

### Samples

In elderly 158 older adult cadavers, 27 hearts were macroscopically assessed for normal aging conditions and selected as study specimens. Exclusion criteria were: structural abnormalities (e.g., cardiac hypertrophy, dilation, and excessive pericardial adipose tissue); moderate and severe arteriosclerosis based on macroscopic and CT imaging observations; and abnormal cardiac weight (<220 g or >400 g for Japanese study population).

After histological reconfirmation, only 23 hearts were considered physiologically representative of an older adult population, excluding CCS pathologic conditions, with age 86.9 ± 7.0 years (72–98 years) and cardiac weight 310.0 ± 34.4 g (239–370 g). Because arteriosclerosis is almost inevitable in this older adult population, hearts with asymptomatic or mild arteriosclerosis were included: An additional 3 intact hearts were used for histologic study of transitional myocytes between the specialized and working myocytes and its extension.

All donors and their families signed informed consent forms to formally donate bodies to Toho University School of Medicine for anatomical education and research use. The protocol for this study was reviewed and approved by Ethics Committee of Toho University Faculty of Medicine (reference number: A20001_A18015_A17033_A17005_25113_23011). All work was confirmed with the provision of the 1995 Declaration of Helsinki (revised in Edinburgh 2000).

### Dissection methods

**Figure 5:**
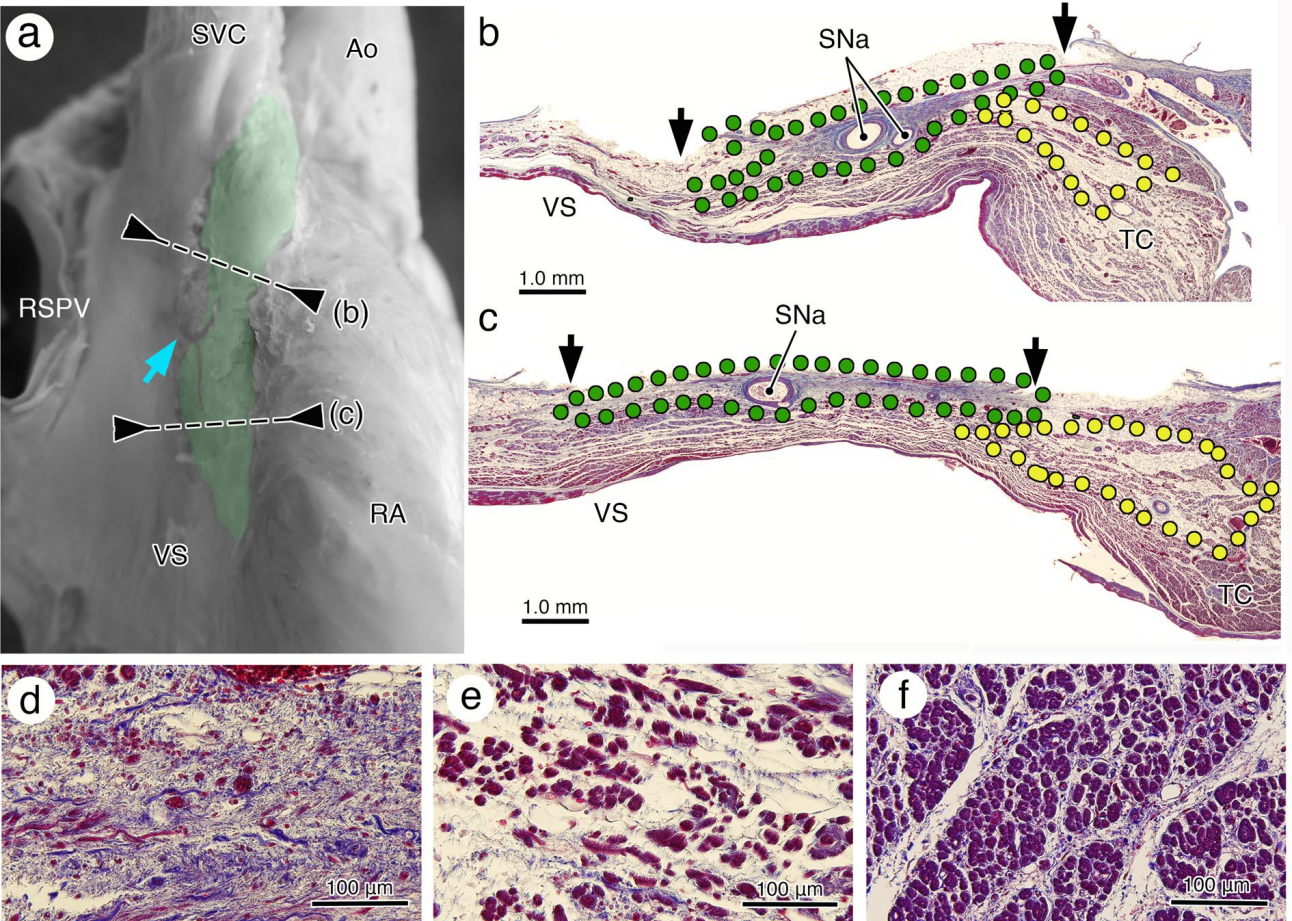
The anatomy and histology of the human sinus node. (**a**) The exposed sinus node, colored in green, after removal of the pericardium and adipose tissues. The sinus node is distinguishable from the surrounding cardiac structures by its location on the terminal groove, yellowish white color, and occasional appearance of the sinus node veins, indicated by blue arrows. The identified sinus node indicated section points for histologic reconfirmation. (**b, c**) The histologic reconfirmation. Sections of the proximal (**b**) and distal (**c**) parts of the exposed sinus node (green dot area and black arrows) with the paranodal area (yellow dot area). The sinus nodal cells (**d**), paranodal cells (**e**), and atrial muscular cells (**f**) are distinct from the cells themselves, extracellular matrix, and their population and size. *Ao* aorta, *RA* right atrium, *RSPV* right superior pulmonary vein, *SNa* sinus node artery, *SVC* superior vena cava, *TC* terminal crest, *VS* venous sinus.

**Figure 6:**
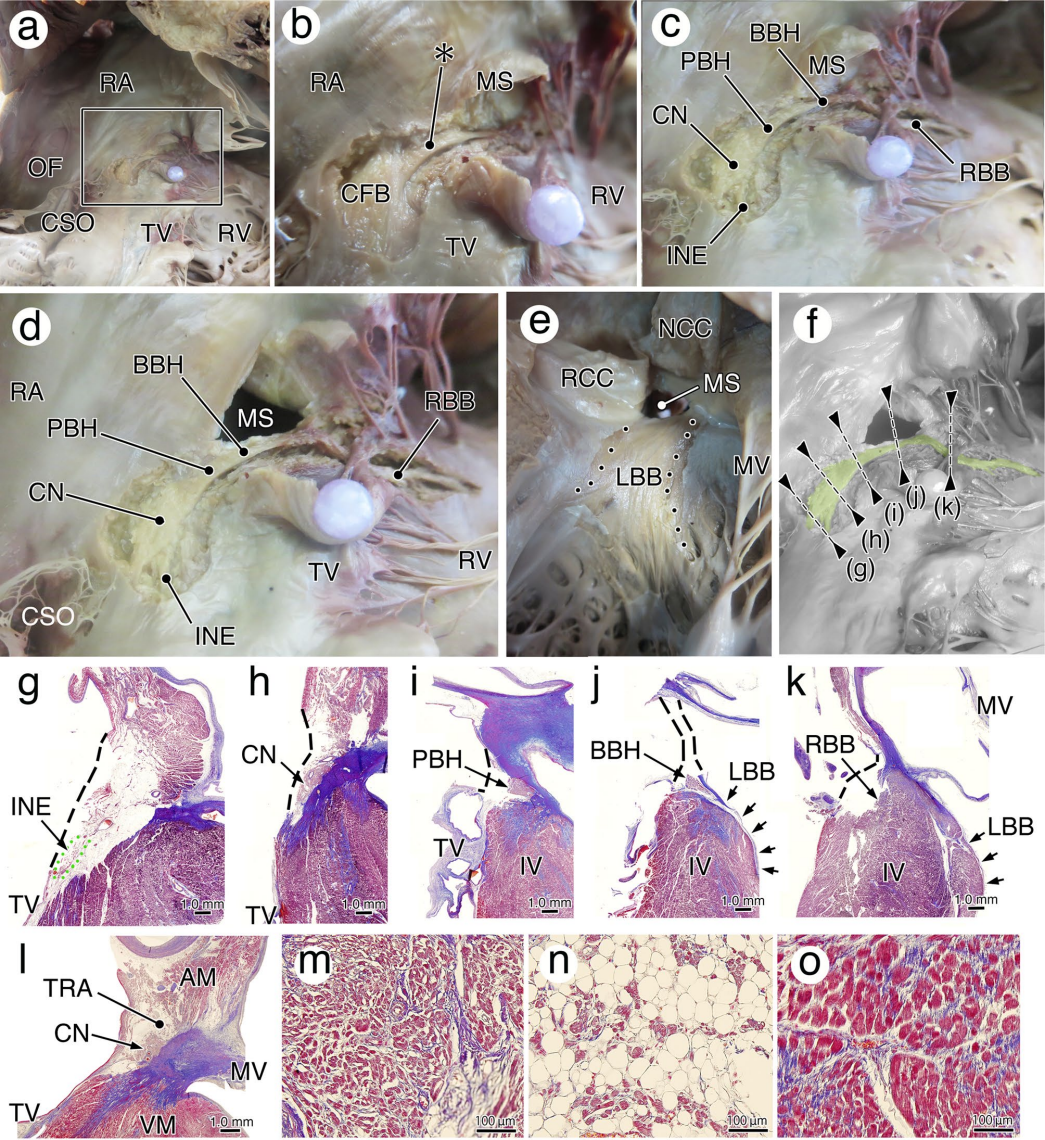
The anatomy and histology of the human atrioventricular conduction system. (**a**–**e**) The dissection steps based on the histologic criteria for identification of the atrioventricular conduction axis (AVCA). The lower (**a**) and higher (**b**) magnification photographs showing the starting dissection point. The expected bundle of His (*, BH) with different muscular arrangement of the ventricle is first detected. (**c**) Relying on continuation with the BH, the compact node (CN) and right bundle branch (RBB) are traced. After cutting off the membranous septum (MS) (**d**), the left bundle branch (LBB) is exposed at the left chamber view (**e**). The exposed AVCA, colored in green (**f**)**,** clearly identified the conduction tissues (**g**–**k**). Black dots show the dissected area. (**l**) The section of heart through the CN shows the different cytological characteristics of the nodal cells (**m**), transitional cells (**n**), and atrial myocytes (**o**). *AM* atrial muscle, *BBH* branching bundle of His, *CFB* central fibrous body, *CSO* coronary sinus orifice, *INE* inferior nodal extension, *IV* interventricular muscle, *MV* mitral valve, *NCC* non-coronary cusp, *OF* oval fossa, *PBH* penetrating bundle of His, *RA* right atrium, *RCC* right coronary cusp, *RV* right ventricle, *TRA* transitional area, *TV* tricuspid valve.

To clarify heart placement within the human body, all hearts were removed after marking the 3D cardiac axes from the intact cadavers. Dissection of the CCS was performed in 2 parts: sinus node (SN) and AV conduction axis (AVCA) distal to the AV node (Figs. 5, 6). Heart stiffening within the surrounding thoracic organs fixed by formalin helps to maintain its in situ morphology, but the CCS dissection may lead to significant transposition of the surrounding structures, including the CCS itself. To minimize the effect of this technical issue, partial bundles of working myocardium were left adhered to the CCS during dissection, so that the CCS was not isolated completely from the working myocardium (Fig. 6a–f). This method results in not only preservation of the absolute CCS localization within the stiffened heart, but also prevents accidental CCS separation from its surroundings during histological preparation of exposed CCS (Fig. 6g–k).

#### Sinus node (SN)

Although the spread of the SN is unclear when many subepicardial adipose tissues are accumulated, this analysis excluded hearts with significant adipose tissue deposition, and SN morphology and dimension were able to be confirmed along the terminal groove. SN veins, running across the superficial part of the SN, are an appropriate macroscopic indicator of the SN versus deep SN artery (a blue arrow in Fig. 5a). When only the epicardium was carefully peeled off and then dissected it under alcoholic storage, the relatively solid SN contained in the fibrous tissue was distinguishable from the soft adipose tissue. The expected SN (Fig. 5a) was histologically reconfirmed as a set of nodal cells surrounded by the fibrous extracellular matrix (Fig. 5b–d). The paranodal cells/area could be recognized histologically in the terminal crest (Fig. 5b,c,e) was completely different with the surrounding atrial myocytes (Fig. 5f), and could not be dissected at a macroscopically. Thus, the identified sinus node represented only the nodal cells packed in fibrous skeleton.

#### AV conduction axis (AVCA)

Based on the histologic criteria of the AVCA, the muscular bundle of the interventricular muscles under the membranous septum of the AV septum were first carefully removed, and the BBH with different colors and muscular fiber directions were exposed from the right chamber side (Fig. 6a,b). Then, relying on the continuity from the BBH, the penetrating bundle of His (PBH) was traced proximally into rigid central fibrous body. Next, the compact AV node was connected extending deeper to the right atrial muscle. The BBH was pursued to the distally to the RBB (Fig. 6c). Thereafter, the membranous septum was removed (Fig. 6d), and the endocardium is carefully peeled from the interventricular muscle from the left chamber side, showing diffuse LBB with a slightly different color is caught (Fig. 6e). The exposed conduction nodes and axis were finally reconfirmed histologically after CT scanning (Fig. 6f); only the confirmed conduction components were used for present data (Fig. 6g–k).

Inferior nodal extensions are sometimes identifiable by compact node continuity (Fig. 6b,j). Similar to the paranodal area around the SN, transitional cells with different extracellular matrix could be confirmed histologically between compact AV node and atrial muscles (Fig. 6l–o). These transitional cells also have a paranodal area, distinct from the surrounding like working myocardium by the cytological and extracytologic matrix properties. This area is difficult to dissect macroscopically because it is contained in loose adipose tissue; therefore, the dissected structure that served as the AV node was only compact node with often-capturable inferior nodal extensions.

All dissection steps were performed with a binocular microscope designed for neurological surgery (*OME5000*, Olympus, Tokyo, Japan) and recorded as digital photographic images by a digital camera (*IXY digital 800IS*, Canon, Tokyo, Japan).

### Histologic reconfirmation

After physical and virtual dissection, histologic reconfirmation of the exposed conduction nodes and axis was performed by Masson trichrome staining on a 6-μm tissue section. Only hearts correctly identified in this final histologic confirmation were used as data. All 23 study cases were confirmed as conduction tissues and did not have a pathologic condition. Data were acquired and reconstructed by a digital microscope system (*Olympus BX51*, Olympus Co., Japan).

### CT imaging

After confirming that the cadaver posture was not twisted and that the thorax was correctly placed anatomically on the dissection bed, the 3 axes of vertical, horizontal, and coronal lines were marked based on dissection bed dimensions and then removed from body.

Next, all 23 cardiac conduction nodes and axis-exposed hearts were scanned by industrial cone-beam CT scanner (*NAOMi-CT* for Industry, the RF, Nagano, Japan) with these parameters: 5 mA, 60–70 kV, and 0.16 mm or 0.08 mm thickness depending on region of interests. To facilitate CCS identification and segmentation in imaging, the exposed CCS was coated as a marker by 10% Iodine solution (Povidone-Iodine, Shionogi Pharmacy Global) and then scanned. These data set were used for main fundamental information including the morphometric analysis.

Then, 11 hearts with exposed CCS were replaced into the body and CT scans were performed to image the human body placement for obtaining conceptional images of the cardiac and CCS within the body as the following procedures. After the thoracotomy, only the anterior pericardium was cut and reflected to leave all chest organs intact, and the heart was removed. After dissection of the CCS, the heart was replaced to the thorax guided by surrounding organs tightly fixed by formalin solution, with the positional relationship and cut vascular plane aligned and then scanned by medical CT scanner (*SOMATOM Emotion 16*; Siemens Healthcare GmbH, Erlangen, Germany) with these parameters: 200 mA, 80–130 kV, 0.75 mm thickness, and 0.2 mm pitch. These data set were used only for conceptional images.

### Imaging data analysis

Image processing and data analysis were performed with commercial DICOM viewer workstations (*ZioCube version 1.0.0.4*, Ziosoft Inc., Tokyo, Japan; *Osirix 64-bit extension version*, Osirix Foundation, Geneva, Switzerland). To obtain theoretical simulation models of CCS transformation from the standing to the lying position of the heart, CT data sets for six standard oblique human hearts with cardiac inclination similar to the mean value were selected from the 23 heart data sets in this study. Using the rotation function of the DICOM viewer software (*ZioCube version 1.0.0.4*, Ziosoft Inc., Tokyo, Japan), each of six standard oblique hearts was reconstructed by rotating all 3D axes (sagittal, frontal, and horizontal) of the heart into five positions of − 2 standard deviation (SD), − SD, ±0, +SD, and +2SD from the mean value for all 23 examined hearts. For these models, the DICOM viewer software (*ZioCube version 1.0.0.4*, Ziosoft Inc., Tokyo, Japan) rotation function was used for CT data set of the standard human CCS.

Morphometric data were obtained by the DICOM 2D or 3D measurement function and reconfirmed by dissected specimens.

## Data Availability

The datasets used in this study are available from the corresponding author on reasonable request.
